# Valuing Human Leptospirosis Prevention Using the Opportunity Cost of Labor

**DOI:** 10.3390/ijerph10051845

**Published:** 2013-05-03

**Authors:** Joseph Arbiol, Maridel Borja, Mitsuyasu Yabe, Hisako Nomura, Nina Gloriani, Shinichi Yoshida

**Affiliations:** 1 Laboratory of Environmental Economics, Graduate School of Bio-resources and Bio-environmental Science, Kyushu University, Fukuoka 812-8581, Japan; E-Mail: oteparbiol@yahoo.com; 2 Department of Epidemiology and Biostatistics, College of Public Health, University of the Philippines-Manila, Manila 1000, Philippines; E-Mail: mpborja@post.upm.edu.ph; 3 Laboratory of Environmental Economics, Department of Agricultural and Resource Economics, Faculty of Agriculture, Kyushu University, Fukuoka 812-8581, Japan; 4 International Education Center, Kyushu University, Fukuoka 812-8581, Japan; E-Mail: hnomura@ agr.kyushu-u.ac.jp; 5 Department of Medical Microbiology, College of Public Health, University of the Philippines-Manila, Manila 1000, Philippines; E-Mail: ninagloriani@yahoo.com; 6 Department of Bacteriology, Faculty of Medical Sciences, Kyushu University, Fukuoka 812-8582, Japan; E-Mail: syn-ichi@bact.med.kyushu-u.ac.jp

**Keywords:** human leptospirosis, contingent valuation method, willingness to contribute to labor

## Abstract

Leptospirosis is a serious public health concern in the Philippines, not only because of its increasing incidence rate, but also because of its significant health and economic impacts. Despite its relatively high seroprevalence, knowledge on the economic burden of disease, particularly on the value that the society places on disease prevention remains limited. Obtaining such information is important within the context of public health policy. This study was conducted in Metro Manila to determine the economic burden of leptospirosis, by asking respondents about their willingness to contribute to labor (*WTC^L^*) for the prevention of leptospirosis. The respondents pledged an average labor contribution of 10.66 h/month. The average *WTC^L^* corresponded to a monetary value of US$4.01 per month when valued using the opportunity cost of labor (leisure rate of time). From the monetized labor contribution, the total economic value of preventing leptospirosis was estimated at US$124.97 million *per annum*, which represents 1.13% of Metro Manila’s gross domestic product (GDP). Estimates from a Tobit regression model identified the respondents’ knowledge regarding leptospirosis, the susceptibility of their homes to flooding, and the proximity of their homes to sewers as significant factors to consider when developing resource contribution programs for leptospirosis prevention. More efforts need to be made in developing community level preventive programs, and in improving public’s knowledge and awareness about leptospirosis.

## 1. Introduction

Leptospirosis is a zoonotic infection caused by spirochetes belonging to pathogenic species of *Leptospira* [1]. Rodents are known to be the principal host of the bacteria, although livestock animals and domestic pets can act as incidental hosts [2]. Human infection occurs from exposure to the infected urine of animal hosts, either by direct contact or via indirect contact with contaminated soil or water [3]. The bacterial infection varies from mild to severe illness, with potentially fatal complications such as jaundice, hemorrhage, and renal dysfunctions [2,4].

Leptospirosis is considered endemic in the Philippines [5]. The leptospirosis incidence rate in the Philippines in 2008 was estimated at 4.8 per million population [6], which can be considered the second highest in Southeast Asia. Leptospirosis outbreaks commonly occur in the urban cities of Metro Manila after heavy rains and floods. One of the notable outbreaks occurred in 2009 after Typhoon Ondoy caused widespread flooding in the metropolis, resulting in 2,089 cases of infection and 162 leptospirosis-related deaths [7]. Recent newspaper and television broadcasts have reported 583 cases with 48 leptospirosis-related deaths from January to November 2011, indicating a 247% increase when compared with the same period in 2010.

Poor sanitation and inadequate garbage disposal are major problems in Metro Manila. Estimates state that of the 6,700 tons of solid waste generated daily in Metro Manila, approximately 6,000 tons is either hauled to the city’s dumpsites or dumped illegally on private lands, in rivers, creeks, bay and vacant sections [8]. Uncollected waste contributes to the epidemiology of leptospirosis by augmenting the proliferation of rats and by further blocking drainage systems, which exacerbates flooding during heavy rains.

Leptospirosis is a public health concern because its increasing incidence rate also imposes health and economic impacts on society. People infected with the disease do not only endure the physical and emotional pain of the illness, but they also have to contend with the financial costs of medical treatments combined with lost work or productivity. Urban slum dwellers often suffer the most during outbreaks because of their precarious living conditions, lack of access to health and sanitation infrastructures, and vulnerability to natural calamities [9]. Furthermore, leptospirosis may pose serious challenges among health authorities to ensure the timely and rapid availability of diagnostic and treatment procedures to patients, given the practical realities of the limited resources and overstretched healthcare system of the Philippines.

Aside from a few serological and epidemiological studies on leptospirosis [4,5,10], there is still a paucity of information and knowledge gaps on the economic burden of leptospirosis in the Philippines, particularly on the value that the society places on disease prevention. The importance of being able to determine the economic burden is that it allows the quantification of costs that could be avoided in reducing the rate of disease incidence. It also enables policymakers to make informed decisions when formulating cost-effective health programs, and to prioritize health investments.

Willingness to pay (WTP) is one method that can be used to measure the economic burden of diseases. It can be derived using a contingent valuation (CV) survey that elicits the maximum amount of money an individual is willing to pay to prevent undesirable health outcomes. The WTP approach is capable of measuring the intangible burden of disease such as pain and sufferings, which are not easily quantified using other valuation techniques [11]. Although the WTP approach is often used to value environmental changes, it has also been applied in the field of health economics to estimate the economic burden of diseases such as Alzheimer’s disease [12], lung cancer [13], influenza [14], malaria [15], and childhood obesity [16].

The use of money as a payment vehicle in CV poses certain limitations, especially when applied within the context of developing countries where many transactions are partially monetized [17], labor markets are imperfect [18,19], and disposable incomes are low [20]. These situations can often impose restrictions on the respondent’s ability to contribute part of their income to the program being valued. Relying on a monetary payment vehicle could lead to an erroneous decision to reject a project that would otherwise be socially viable, particularly in poor regions where WTP is low due to the aforementioned limitations confronting the respondents. Consequently, non-monetary payment in the form of labor contribution is often used as an alternative utility measure in CV [17,18,19,20,21]. This approach has gained higher acceptance among respondents in developing countries because it is able to capture those economic activities and limitations outside the scope of a monetary mechanism.

This paper describes the initial exploration of an on-going project related to the economic burden of disease in the Philippines. It represents the first attempt to measure the economic value of preventing leptospirosis among urban dwellers in Metro Manila using willingness to contribute in terms of labor (*WTC^L^*) approach. The use of the *WTC^L^* approach has particular significance in the study considering that the groups vulnerable to leptospirosis are those living in the poor urban communities of Metro Manila, where poverty incidence is high and unemployment prevails. Thus, using money as a payment vehicle in CV may prevent them from fully expressing their true appreciation of the proposed prevention program. Moreover, the valuation scenario requires the engagement of the respondents in the proposed activity of the leptospirosis prevention program. The factors affecting respondents’ *WTC^L^* for the prevention of leptospirosis are also presented in this paper.

The remainder of the paper is organized as follows: in the next section, the contingent valuation survey and analytical framework are discussed. The empirical results of the econometric model are then presented. The policy and methodological implications are then discussed, and finally conclusions are drawn.

## 2. Methods

### 2.1. Contingent Valuation (CV) Survey

The University of the Philippines-College of Public Health (UP-CPH) initially conducted a seroprevalence survey from October 2010 to January 2011 to determine the prevalence of leptospirosis in Metro Manila. Using a three-stage cluster sampling design, the seroprevalence survey collected blood specimens from 1,136 individuals representing 413 households across 30 *barangays* (the term *barangay* refers to a village and represents the lowest geopolitical subdivision in the Philippines) in the said area. The blood specimens of the respondents were tested for anti-*Leptospira* antibodies using an Enzyme-linked Immunosorbent Assay (ELISA). Likewise, information on the socio-demographics, knowledge and awareness about leptospirosis and home-environmental attributes of the selected respondents were also collected. The respondents were also provided with health information about leptospirosis after the data collection. At the end of the seroprevalence survey, a total of 839 individuals were identified seronegative in the ELISA. The 839 seronegative individuals were followed-up after a few months to determine the incidence of sero-conversion, however only 447 individuals representing 199 households agreed to participate in the second blood screening.

The contingent valuation (CV) survey was conducted from May to June 2011. The CV survey was approved by the ethics committee of the UP-CPH as part of the burden of leptospirosis study. Based on the list of 199 households that participated in the sero-conversion screening, only one respondent per household was selected for the CV survey. The eligible respondents were those who agreed to participate in both the first and second blood screenings, and only those aged 15 or over in order to comply with the country’s law on legal working age. During field interviews in the CV survey, a higher response was generated among females probably because they usually stayed home to take full responsibility for housework. This situation reflects the characteristics of the seroprevalence survey, where the respondents for the CV survey were drawn. The prevalence of leptospirosis based on the detection of anti-*Leptospira* antibodies among the CV respondents was estimated at 24.38%. However, it should be noted that although these respondents had anti-*Leptospira* antibodies, they were all asymptomatic and did not have clinical manifestations of leptospirosis.

The CV questionnaire was developed after taking into account the inter-related problems of indiscriminate dumping of garbage, rat proliferation, and leptospirosis outbreaks in Metro Manila. The questionnaire contained a hypothetical leptospirosis prevention program that required the local community to participate and contribute some of their time in voluntary work for the prevention of leptospirosis. The tasks included environmental cleanup activities to eradicate rats in their respective *barangay* and health advocacy activities. The questionnaire was pretested to see how it works and to make necessary changes before the start of the actual survey.

The research staff of the UP-CPH conducted face-to-face interviews with the respondents. While respondents were likely to have already developed familiarity with leptospirosis considering that they had previously participated in the seroprevalence survey, the research staff still reminded them on the causes, clinical symptoms of infection and risk of acquiring leptospirosis prior to interview. The respondents were also informed about all aspects of the study and gave written informed consent. During the interviews, the respondents were presented with a detailed description of the goods being valued, and the method of their contributions. Using an open-ended CV format, the respondents were initially asked about the maximum amount of time that they would be willing to contribute in terms of voluntary labor to the proposed leptospirosis prevention program. The respondents were asked a further question at the end of the interview regarding their expected wages if they were to get paid for the voluntary work. The expected wage rate was used to calculate the opportunity cost of their labor contribution to the proposed program.

In the analysis, the respondents’ responses to the CV questions were matched with their corresponding data taken from the previous seroprevalence survey, which includes: information on the socio-demographics of the respondents in both individual and household levels, respondents’ knowledge and awareness about leptospirosis and the environmental attributes of the respondents’ homes. Data regarding the flood susceptibility index was derived by plotting the residential address of each respondent on an interactive GIS of the flood and landslide susceptibility map for Metro Manila provided by the Mines and Geosciences Bureau [22]. Data from six respondents were removed from the analysis due to missing values.

### 2.2. Analytical Framework

#### 2.2.1. Willingness to Contribute to Labor (*WTC^L^*) for Leptospirosis Prevention

An individual’s WTP for goods and services is typically elicited in monetary units. However, recent studies have shown the plausibility of using labor contribution as a payment vehicle in CV surveys. For instance, labor contribution has been used to measure WTP for potable drinking water [17]. Other studies have adopted money and labor contributions to value tsetse fly and trypanosomosis disease control [18,19], forest fire prevention [21], and marine resources [20]. These studies have shown a higher level of acceptability regarding labor contribution as a payment vehicle in CV studies within the context of developing countries, where labor markets are imperfect and low-income or partially monetized economies predominate.

The present study adopted the *WTC^L^* approach after taking into account the socio-economic characteristics of survey respondents. Many of the respondents live in Metro Manila’s poor urban communities, and they have incomes and resources that are often insufficient to meet the basic standard of living. In addition, in Metro Manila some workers’ wages are paid partly in money and the remainder in a non-monetary unit, for instance with food. The presence of tight income constraints and labor market imperfections could prevent individuals from fully expressing their true thoughts regarding the program when monetary payment is used to measure their WTP. Under these situations, a welfare measure that relies on monetary WTP may not only lead to the possible rejection of socially viable programs, but it may also undermine the potential contributions and concerns of low income societies.

The theoretical framework of using time as a payment vehicle for CV studies is based on the notion of a time-compensating surplus, which is analogous to a compensating surplus measure of monetary WTP [23]. Based on this framework, welfare estimates of preventing leptospirosis can be expressed as:

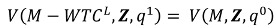
(1)


The indirect utility function (*V*) denotes the amount of time a respondent is willing to contribute to labor (*WTC^L^*) for the proposed leptospirosis prevention program, and can be utilized to provide a change in environmental quality, from an environment prone to leptospirosis (*q^0^*) to an improved environment that is less prone to leptospirosis (*q^1^*). The labor time contribution to the proposed program represents a payment and involves a reduction in the respondents’ time budget (*M*) available for other activities (work or leisure). The variable (*Z*) represents the vectors of the socioeconomic variables that vary across the respondents.

CV surveys have different elicitation formats that take the forms of open-ended questions, discrete choice, and payment cards. In this survey, because of the limitation of the sample size, an open-ended CV format was used to elicit each respondent’s *WTC^L ^*for leptospirosis prevention. A common drawback of this format is the generation of a considerable number of zero responses [24]. Hence, a linear specification of the model was performed using Tobit regressions to take into account the difference between censored (zero) and continuous observations in the process of estimation, leading to more consistent parameter estimates [25]. The Tobit regression can be generally expressed as follows [26]:

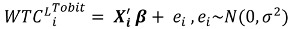
(2)
where 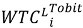 is an unobserved continuous dependent variable,* Xi'* is a vector of explanatory variables, β is a vector of coefficients, *e_i_* is an independently distributed error term assumed to be normal with a zero mean and constant variance σ^2^*,* and *i =* 1, 2, …, n denotes individuals in the sample. The observed *WTC^L^* variable takes the following form:

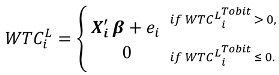
(3)


The obtained *WTC^L^* values for the proposed leptospirosis prevention program, including auxiliary data regarding the respondents’ socioeconomic and environmental characteristics, were incorporated in the Tobit regression model. An empirical model was then derived for the determination of factors affecting *WTC^L^* for the prevention of leptospirosis. The empirical model is presented as:

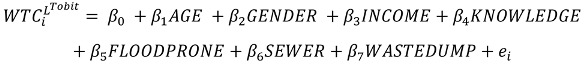
(4)
where β_0_, β_1_, …β_7_ are coefficients and *e_i_* is the normally distributed error term. The dependent and independent variables are defined in Table 1. This study hypothesized that the *WTC^L^* in a leptospirosis prevention program is determined by socio-economic factors related to the respondents’ age, gender, income, and knowledge about leptospirosis, as well the environmental characteristics of the respondents’ homes in terms of susceptibility to floods and proximity to sewers and waste dumpsites.

**Table 1:** Variable description and sample statistics (n = 193).

Variable	Description	Mean	Std. Dev.	Min	Max
**1. Dependent**					
*WTC^L^*	Labor contribution of the respondents to have the proposed program implemented (in hour/month)	10.66	22.35	0	160
**2. Independent**					
*Socio-economic Attributes*					
*AGE*	Age of the respondent (in years)	37.72	13.03	15	64
*GENDER*	1 if the respondent is male; 0 if female	0.34	0.48	0	1
*INCOME*	Monthly income per household size (in ‘000 pesos)	0.78	1.84	0.4	17.5
*KNOWLEDGE*	1 if the respondent’s knowledge about leptospirosis is high (test score≥70%); 0 if otherwise	0.55	0.50	0	1
*Environmental Attributes*					
*FLOODPRONE*	1 if the respondent's house location is highly prone to flooding; 0 if low	0.56	0.50	0	1
*SEWER*	1 if the respondent's house location is near (about 10 meters) to a sewer; 0 if far	0.90	0.29	0	1
*WASTEDUMP*	1 if the respondent’s house location is near (about 10 meters) to a waste dumpsite, 0 if far	0.13	0.34	0	1

#### 2.2.2. Converting Labor Contribution into Monetary Value

Respondents who did not accept the hypothetical scenario described in the CV survey were treated as having zero values, while respondents with positive responses had their labor contribution converted into monetary values. The assumption is that time to be spent in doing voluntary labor for leptospirosis prevention will require an opportunity cost, hence it has economic value. To covert *WTC^L^* into monetary value, the respondents were asked about their expected wage rate (pesos/hour) if they were to get paid for the voluntary labor. The expected wage rate was used to estimate the opportunity cost of time contribution for leptospirosis prevention program. The opportunity cost of time may vary considerably from one respondent to another, and depends on whether working time or leisure is to be allocated for labor contribution to the leptospirosis prevention program. If respondents are making trade-off between working time and time spent towards contributing to leptospirosis prevention, then the opportunity cost of time can be valued in a relatively straightforward manner using the average wage rate of time (*WRT*) as defined in Equation (5):

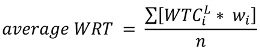
(5)
where 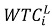 represents willingness to contribute to labor for leptospirosis prevention by respondent *i*; *w_i_* represents the expected wage of respondent *i*; and *n* represents the total number of respondents in the sample. However, if leisure time is being substituted for time spent contributing to leptospirosis prevention, then the opportunity cost of time can be valued using the leisure rate of time. Based on the method developed by Cesario (1976) as cited in O’Garra (2009), the average leisure rate of time (*LRT*) is equivalent to a third of the value of the average wage rate of time [27]:

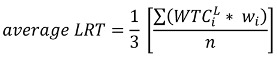
(6)


## 3. Results

### 3.1. Characteristics of the Respondents

Table 1 provides the summary statistics of the socio-economic and environmental attributes of the respondents for this study. The average age of the respondents was 37.82 years old. Approximately 66% of the respondents were female and 34% were male, and more than half of the respondents were unemployed. Respondents who were employed were engaged mostly in sales-related work, transport services, personal care and services, agriculture and construction, food production and services, management, grounds care and maintenance, and administrative and health support services. The monthly income per household size ranged between 400 pesos to 17,500 pesos (US$10 to US$406). Fifty five percent of the respondents exhibited a high level of knowledge and awareness regarding the cause of the disease, mode of transmission, signs and symptoms, and the prevention and treatment of leptospirosis. The homes of the respondents were predominantly located within high-risk flood areas (56%) and near open sewers (90%). Only a small number of respondents (13%) lived near waste dumpsites.

### 3.2. Labor Contribution for Leptospirosis Prevention

When respondents were asked about the amount of time they were willing to contribute for voluntary labor to the proposed leptospirosis prevention program, the results indicated a broad acceptance of labor as a payment vehicle. As shown in Table 1, the distribution of labor contributions ranged from 0 to 160 h/month. The average labor contribution was 10.66 h/month. Out of the 193 respondents, about 60% (n = 115) were willing to contribute in terms of labor for leptospirosis prevention program, while the remaining 40% (n = 78) gave a zero value. It was noted during the interview that the reasons often cited for refusing to participate in the proposed prevention program included being too busy with existing jobs, lack of concern about leptospirosis, and the belief that government should pay for the program.

### 3.3. Monetary Value of Preventing Leptospirosis

The opportunity cost of time that respondents are willing to contribute toward leptospirosis prevention was valued at 507.25 pesos (US$12.03) a month or 6,087 pesos (US$144.33) *per annum* based on the average wage rate of time. The average leisure rate of time, which represents a one-third value of the average wage rate of time, was estimated at 169.08 pesos (US$4.01) a month or 2,027 pesos (US$48.09) *per annum*. To further estimate the total benefit of preventing leptospirosis, the estimated annual opportunity cost of time (wage rate and leisure rate) was multiplied to the estimated 2,601,094 households living in Metro Manila [28]. A total annual benefit of 15.83 billion pesos (US$375.28 million) was derived when using the wage rate of time, while 5.27 billion pesos (US$124.97 million) was estimated using the leisure rate of time. These values represent 3.40% (wage rate of time) and 1.13% (leisure rate of time), respectively, of the annual GDP of Metro Manila, valued at 465.7 billion pesos or equivalent to US$10.57 billion [29]. To avoid overestimation, this study opted to use 1.13% of Metro Manila’s GDP to value the leptospirosis prevention program.

### 3.4. Factors Affecting WTC^L^ for Leptospirosis Prevention

Table 2 presents the results of the Tobit regression analysis of the factors affecting *WTC^L^* for the prevention of leptospirosis. The regression model performed adequately with a chi-square value of 13.33, which was significant at a *p*-value of 0.05. The regression model identified *KNOWLEDGE, FLOODPRONE*, and *SEWER* as statistically significant factors affecting *WTC^L^* with *p*-values less than 0.10 and 0.05. The *WTC^L^* was positively associated with the respondent’s knowledge about leptospirosis and with respondents living in high-risk flood areas. In contrast, *WTC^L^* was negatively associated with respondents living near sewers. Other variables such as *AGE*, *GENDER*, *INCOME*, and *WASTEDUMP* were not statistically significant (*p*-value > 0.10) in explaining the dependent variable.

**Table 2:** Results of Tobit regression analysis of factors affecting *WTC*^L^ for the prevention of leptospirosis.

Variable	Coeff.	Std Err.	*t*-value
*AGE*	−0.043	0.200	−0.22
*GENDER*	3.489	5.719	0.61
*INCOME*	−1.220	1.394	−0.88
*KNOWLEDGE*	9.289 *	5.095	1.82
*FLOODPRONE*	10.613 **	5.262	2.02
*SEWER*	−20.063 **	8.112	−2.47
*WASTEDUMP*	7.840	7.490	1.05
Constant	7.634	11.625	0.55
Log-likehood	−588.845		
*X^2^*	13.33 *		

* Significant at the 10% level, ** significant at the 5% level.

## 4. Discussion

The number of leptospirosis outbreaks in Metro Manila has been directly attributed to socio-economic and environmental factors including unplanned urbanization and growth in urban slums, poor waste disposal, poor sanitation, rat infestation, heavy rains, and floods [4]. It is expected that the intensity of the disease incidence and the magnitude of leptospirosis outbreaks will continue to increase as extreme weather events like typhoons which occur very frequently in the Philippines. While current leptospirosis preventive initiatives of the Philippine government have focused mainly on providing health advisories during monsoonal rains, developing rapid diagnostic test, and providing post-exposure treatment by prophylactic medication, it would also be essential to develop and implement a broad-based leptospirosis prevention strategy involving community to engage in effective actions against leptospirosis for which at present, none is available. As indicated by the results of our study, there is a positive public support towards community level leptospirosis prevention activity considering that the majority of the respondents are willing to participate and contribute their time to the proposed prevention program.

The study also gave a clear indication that respondents’ knowledge about leptospirosis, susceptibility of home to flooding, and proximity of home to sewers are significant determinants of willingness to contribute in terms of labor for the proposed leptospirosis prevention program. The positive association between *WTC^L^* and *KNOWLEDGE* suggests that respondents with a higher knowledge regarding leptospirosis placed a higher value on disease prevention programs. This result is consistent with prior studies found in the literature. Pokou *et al*. found that willingness to contribute to labor for tsetse control in Africa is positively associated with individual’s knowledge about disease [30]. Van Benthem *et al*. also confirmed that individuals with greater knowledge about dengue fever were more likely to engage in preventive measures than individuals with no knowledge [31]. Thus, it is likely that respondents with a high level of knowledge about leptospirosis are more informed of the potential effects of the disease on their lives, and thereby more willing to pursue healthy behavior or activities that would prevent them from contracting the disease.

It should be noted that although more than half of the respondents (55%) demonstrated high levels of knowledge and awarness of the cause, mode of transmission, signs and symptoms, and ways to prevent leptospirosis; a substantial proportion of respondents (45%) demonstrated a moderate though inadequate level of knowledge about leptospirosis. Considering the importance of knowledge in disease prevention, this study supports the need to improve peoples’ knowledge via education and health promotion programs on leptospirosis.

Studies in the literature have associated flooding with numerous outbreaks of leptospirosis around the world [32,33,34,35]. A more recent study further confirmed that the increased risk of acquiring anti-*Leptospira* antibodies is significantly associated with various environmental attributes including exposure to floods [36]. These findings may offer an explanation for the positive association between *FLOODPRONE* and *WTC^L^*. Respondents living in Metro Manila’s high-risk flood areas were more willing to contribute greater hours to the prevention of leptospirosis because they probably perceived themselves to be at high risk of infection.

Sewers often serve as habitats for rats. Rat urine and feces are likely to contaminate these sewers, thereby increasing the risk of disease exposure to nearby dwellers. Such a premise was considered in this study, which made us expect a positive association between *WTC^L^* and *SEWER* (the proximity of homes to sewers)*.* Contrary to our expectations, our finding showed that respondents living near sewers had lower *WTC^L^* values compared with those living far from sewers. There are possible explanations for this finding. First, we could relate this finding on the recent study that shows no statistically significant correlation between the seroprevalence of leptospirosis in Metro Manila and proximity homes to open sewers [37]. It is possible that respondent living near the sewer may feel that they are not at risk of leptospirosis, hence are less likely to contribute their time to the proposed disease prevention program. Second, people living near to sewers are intrinsically associated with urban slums and poverty [38,39]. People living in urban slums usually have temporary homes, and in most cases illegal with uncertain tenure. Several studies have confirmed the significant and positive impact of tenure security on the willingness to participate in collective action for urban slum services [40,41]. Tenure security creates incentives for individual to participate in collective action because the resulting improvements can be capitalized and the anticipated benefit for service provision is accrued over a longer period [40]. Hence, we speculate that respondents living near sewers may have to contend with the constant threat of possible evictions, which may hinder them to capitalize on the disease prevention scenario (environmental clean-up) because of the anticipated benefits pay back only in the long-term. Further, it is also possible that respondents living near to sewers are facing extreme poverty. They are less likely to spend time volunteering because they are too concerned about their survival, and the time spent on participation could be used as labor for cash income elsewhere. While our assumptions may imply that disease prevention and health promotion measures should go hand in hand with improvement in the housing and living standards of the respondents, a more detailed research on understanding the motivations shaping community participation in disease prevention is necessary to verify our findings.

The willingness to pay (WTP) approach is becoming increasingly accepted in health economics to elicit utility values for health states. It poses certain advantages and disadvantages when compared with other health valuation methods. Unlike the adjusted life years (QALYs) and disability-adjusted life years (DALYs) methods, where health values are indexed on a 0 to1 scale of health [42], the WTP approach provides a direct measure of benefits of public health interventions in monetary units which can be easily compared to cost of a policy, thereby enabling policymakers to make straightforward decision rule of adopting health intervention that maximizes net benefits [43]. In contrast to DALYs, where disability values are based on health experts’ perceptions of the level of well-being associated with various conditions [44], WTP values may provide a meaningful measure of changes of health states considering that it represents the preferences of individuals affected by the disease. While WTP approach shows similarity with QALY in terms of representing individual preferences, it is less restrictive and allows the possibility that preferences over health outcomes depend on individual characteristics, such as wealth and baseline risk [43]. However, estimates from WTP could have substantial variation between individuals because it imposes few constraints on individual preferences [43,45]. Further, extraneous influences from imperfect labor markets, presence of partially monetized transactions, and income asymmetries can cause monetary WTP estimates to be problematic and possibly ignore the potential contributions of individuals with lower incomes [18,19,20].

This study attempted to use labor-time contribution as payment vehicle to WTP for disease prevention, and to monetize labor-time contribution using the opportunity cost of time in order to capture some of the limitations of monetary WTP approach within the context of low income households. Although results from this study reveal that our methodological approach ought to be considered when applying WTP in health valuation studies, further improvements are required to deal with several limitations.

First, the average *WTC^L^* may just represent the value that poor urban dwellers in Metro Manila place on leptospirosis prevention considering that the CV respondents were mostly low-income earners. The value that high-income earners place on leptospirosis prevention is underrepresented in the study. Future work could compare the value of leptospirosis prevention and preference for labor contribution between high and low income earners in Metro Manila should be considered in future studies. This could be done through a wider randomized survey to include respondents within the high-income brackets of the metropolis.

Second, the presence of zero responses is a relatively common observation in CV surveys. The proportion of zero responses can range from 24% to 60% [46,47]. Although the proportion of zero responses in this study is within the acceptable range identified in the literature, a more detailed examination on the factors that motivate zero bidders may be useful in understanding their concerns and attitudes toward the valuation method.

Third, the economic value of disease prevention may vary based on the individual’s experience with the disease. It is possible that the *WTC^L^* of an individual who had experienced leptospirosis might be higher when compared with someone who has no experience with disease. Hence, a comparison of the *WTC^L^* values between leptospirosis patients and non-patients may merit further research.

Fourth, the used of open-ended elicitation format in our study may have caused some variations in the values of *WTC^L^* given the presence of a number of zero responses and few high *WTC^L^* values (maximum 160 h/month). Open-ended elicitation format suffers from lack of incentive compatibility and may encourage respondents to free-ride [48]. The use of dichotomous elicitation format could be explored in future studies considering that it is more demand revealing and is less cognitively burdensome to comply with [48,49]. It is also possible that respondents might find time constraint difficult to capture as compared with budget constraint, therefore explicitly reminding the respondents about their time constraint during interviews may help in improving their responses.

Finally, this study did not take into account the substitution between labor and money contributions as payment vehicle for CVM. Future research might consider examining the determinants of willingness to contribute to the disease prevention in relation to the form of contributions, in particular money, labor or a mix of both.

## 5. Conclusions

The main purpose of this study was to measure the economic welfare of preventing leptospirosis in an urban setting without reliance on the conventional contingent valuation method (CVM). Instead of asking for monetary payment, individuals were asked how much time they would be willing to contribute to the proposed leptospirosis prevention program. Such an approach is useful not only within the perspective of poor economies, but also in situations where community engagement is important for the successful implementation of development programs.

This study shows that CVM payment in the form of labor contribution was acceptable among the urban dwellers in Metro Manila. The importance of using this method becomes more apparent when time contributions are valued using the opportunity cost of labor. Using a leisure rate of time, the derived aggregate estimate of the total benefit of leptospirosis prevention valued at US$124.97 million *per annum* provides a solid ground for investment in preventive measures. Knowledge about leptospirosis, susceptibility of home to flooding, and proximity of home to sewers are key factors to consider when developing resource contribution programs for the prevention of leptospirosis. More efforts need to be made in developing community level preventive programs, and in improving the knowledge and awareness of the public about leptospirosis. Lastly, while the technique used in the study has certain limitations, it still affords a modest contribution to the limited literature on the economic burden of leptospirosis, and on the use contingent valuation method in valuing health program benefits within the context of developing countries.

## Appendix: CVM Questionnaire

**Scenario:** Assuming that a program for the prevention of leptospirosis is being implemented. The program includes activities related to environmental cleanups and health advocacy activities for leptospirosis prevention. People are encouraged to participate in the proposed program. However, their participation will be strictly on voluntary basis.

### Questions: Labor Time Contribution

G1. Would you be willing to support the program as a volunteer?

  □1. Yes OR □2. No

G2. If, YES, how much time are you willing to give in voluntary work in support of the program?

  Hours per day [ ______________ ]

  Days per week [ ______________ ]

  How many weeks? □1. [ _______ ] number of OR □2. every week

G3. If you are to get paid for voluntary labor, what is your expected wage?

  Respondent: [ _______ ] pesos/hour
